# DAM SRAM CORE: An Efficient High-Speed and Low-Power CIM SRAM CORE Design for Feature Extraction Convolutional Layers in Binary Neural Networks

**DOI:** 10.3390/mi15050617

**Published:** 2024-04-30

**Authors:** Ruiyong Zhao, Zhenghui Gong, Yulan Liu, Jing Chen

**Affiliations:** 1 Shanghai Institute of Microsystem and Information Technology, Chinese Academy of Sciences, Shanghai 200031, China; zry@mail.sim.ac.cn (R.Z.); zhenghuig@mail.sim.ac.cn (Z.G.); ylliu@mail.sim.ac.cn (Y.L.); 2 University of Chinese Academy of Sciences, Beijing 100049, China

**Keywords:** in-memory computing, SRAM, self-stabilizing voltage, ultra-low power

## Abstract

This article proposes a novel design for an in-memory computing SRAM, the DAM SRAM CORE, which integrates storage and computational functionality within a unified 11T SRAM cell and enables the performance of large-scale parallel Multiply–Accumulate (MAC) operations within the SRAM array. This design not only improves the area efficiency of the individual cells but also realizes a compact layout. A key highlight of this design is its employment of a dynamic aXNOR-based computation mode, which significantly reduces the consumption of both dynamic and static power during the computational process within the array. Additionally, the design innovatively incorporates a self-stabilizing voltage gradient quantization circuit, which enhances the computational accuracy of the overall system. The 64 × 64 bit DAM SRAM CORE in-memory computing core was fabricated using the 55 nm CMOS logic process and validated via simulations. The experimental results show that this core can deliver 5-bit output results with 1-bit input feature data and 1-bit weight data, while maintaining a static power consumption of 0.48 mW/mm^2^ and a computational power consumption of 11.367 mW/mm^2^. This showcases its excellent low-power characteristics. Furthermore, the core achieves a data throughput of 109.75 GOPS and exhibits an impressive energy efficiency of 21.95 TOPS/W, which robustly validate the effectiveness and advanced nature of the proposed in-memory computing core design.

## 1. Introduction

**Figure 1:**
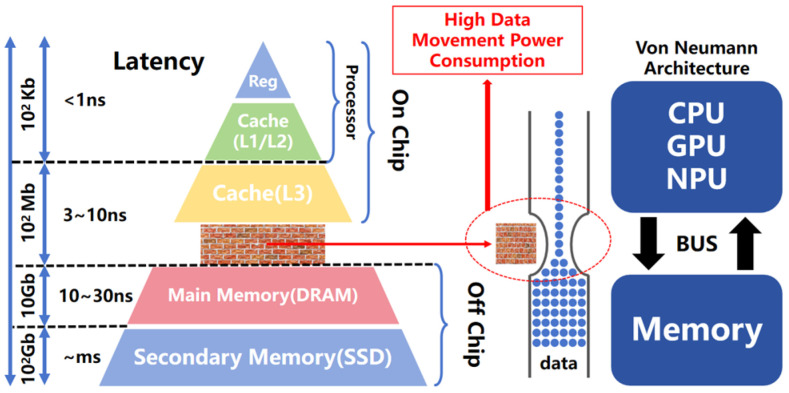
The “Memory/Power Wall” bottlenecks of the Von Neumann architecture. The Von Neumann bottleneck introduces substantial power consumption and transmission latency, which are caused by the movement of data.

At present, neural networks face significant challenges pertaining to the storage capacity and computational energy consumption of mobile devices, especially within resource-constrained and power-sensitive environments. Owing to advancements in AI technology, the storage requirements and computational intensity of complex models such as Convolutional Neural Networks (CNNs) and Deep Neural Networks (DNNs) have increased significantly, leading to substantial memory occupancy due to an abundance of parameters and frequent data transfers; these, in turn, lead to the expenditure of considerable quantities of energy. As depicted in Figure 1, when executing the Multiply–Accumulate (MAC) operations characteristic of CNNs or DNNs, the conventional Von Neumann architecture [1] requires data to be fetched from the memory to the Central Processing Units (CPUs) or Graphics Processing Units (GPUs), thereby increasing the latency and power consumption [2,3,4]; this limits the real-time performance and sustained operation capabilities of these models on mobile platforms.

Neural network models may encompass millions or even billions of parameters, not only occupying vast amounts of memory space but also generating frequent and substantial demands for data migration during read, computation, and update processes. Furthermore, traditional CPU- or GPU-based computing architectures are not inherently well suited to the parallelism and high computational density of deep learning tasks, with power efficiency issues being particularly pronounced when handling intricate Convolutional Neural Networks and Deep Neural Networks. Even with the deployment of low-power specialized hardware accelerators, without effective strategies for the optimization of energy efficiency, the problem of energy loss incurred by the extensive movement of data during large-scale computations remains inadequately addressed.

In response to the pressing demand for storage resources and energy consumption presented by contemporary neural network computations, particularly the “power wall” and “memory wall” [5] challenges encountered in mobile device applications, this paper introduces a novel DAM SRAM in-memory computing core intellectual property (IP). This IP integrates the storage capability of a 6T SRAM cell with the computational prowess of a 5T dynamic aXNOR structure, thus effectively performing single-bit multiplication tasks. To mitigate the issue of high power consumption during large-scale parallel computations, it specifically incorporates a dynamic aXNOR computing mode (aXNOR-based computing involves analog current-based bitwise XOR operations within units, which substitutes unit-level multiplications with XORs, simplifying unit design; current computation is used because, per Kirchhoff’s laws, current accumulation in a bus adds up individual unit results, facilitating MAC operations within the array), which reduces both the static and computational power consumption of the DAM SRAM core.

**Figure 2:**
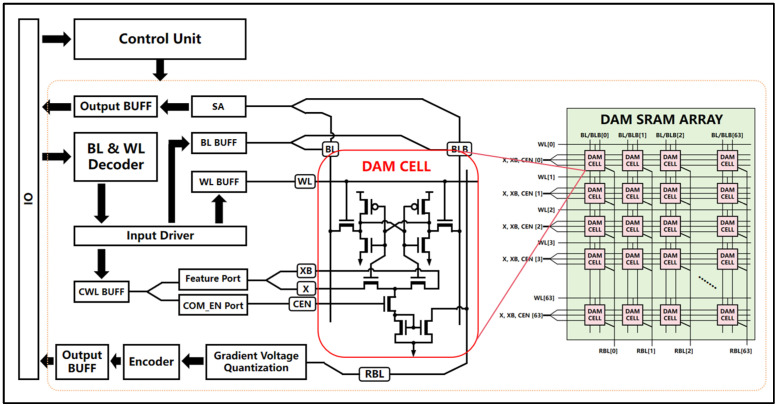
The overall macroscopic structure of the DAM SRAM computational module comprises several integral components: a 64 × 64 array of DAM SRAM cells, 64 Gradient Voltage Quantization and Encoder (GVQE) circuits distributed along the column lines, a control unit, decoder circuits for address translation, driver circuits, and input/output buffer (BUFF) circuits, among others.

As illustrated in Figure 2, the proposed DAM SRAM core’s overall architecture comprises a 64 × 64 in-memory computing array (DAM SRAM ARRAY), 64 Gradient Voltage Quantization and Encoder circuits distributed per column, a control unit, decoder circuitry, driver circuits, and input/output buffers. Fabricated using SMIC 55 nm process technology, it exhibits a superior energy efficiency of 21.95 TOPS/W, a data throughput of 109.75 GOPS, and an output precision of 5 bits while operating at a frequency of 500 MHz. This design successfully maps various algorithmic steps of binary neural networks onto the in-memory computing architecture, thus significantly reducing the overheads of DSP circuits and optimizing the cell area and layout in order to increase the integration density and energy efficiency ratio. The contributions of this work are summarized as follows: (1) The development of an innovative 11T SRAM cell that integrates storage and computational functionalities, enables large-scale parallel Multiply–Accumulate (MAC) operations to be performed within a memory SRAM array, and achieves ahigh utilization of area and a compact layout; (2) the implementation of a dynamic aXNOR computation mode that drastically decreases both the computational and static power consumption of the array; and (3) the design of a voltage gradient quantization circuit that is able to enhance the operational accuracy.

## 2. Design and Analysis

### 2.1. The 11T DAM SRAM Cell

**Figure 3:**
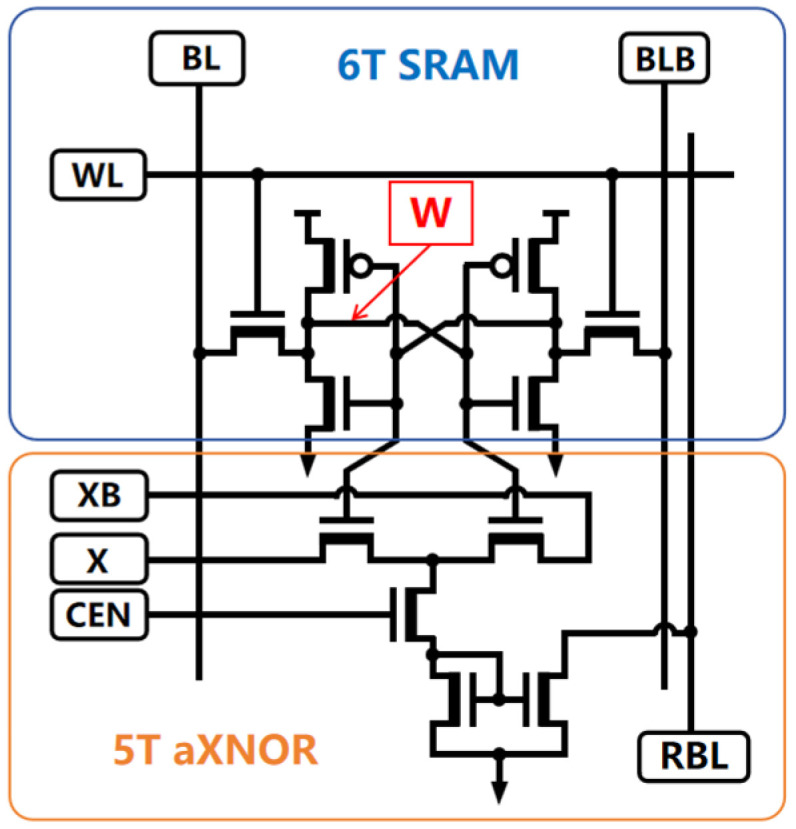
The DAM SRAM bit cell integrates the storage capability of a 6T SRAM cell with the computational prowess of a 5T dynamic aXNOR structure, effectively performing single-bit multiplication tasks.

As shown in Figure 3, in the proposed DAM SRAM cell design, the 5T aXNOR structure achieves physical separation between the computation and data storage, while maintaining logical synergy. The inclusion of computation transistors generates analog computation currents, denoted as unit current IMAC, which do not engage in any direct signal interaction or electrical pathway interference with the original data stored in the conventional 6T SRAM cell. During high-speed computational cycles, there is no electrical coupling effect between the computation transistors executing the computational tasks and the storage transistors holding the data; this ensures that fluctuations in the current generated by the computation transistors cannot propagate through word lines or bit lines into the storage region. Consequently, this design effectively prevents unintended state flips from occurring in the data in neighboring storage cells due to transient voltage variations on the word or bit lines, thereby fundamentally avoiding the appearance of unnecessary data write errors caused by potential interference factors during word line read operations.

Under normal operations, the 11T SRAM cell benefits from its unique decoupled design of storage word lines and computation word lines, enabling dual-mode functionality: the standard SRAM storage mode and computation mode. In the traditional SRAM storage mode, the 11T SRAM cell retains the fundamental storage and retrieval characteristics of a 6T SRAM unit, storing critical weight information W in a binary form for neural networks or other data-intensive applications. This ensures that these weights can be accessed and updated rapidly and reliably in response to read requests from a host processor or associated computational components.

In the computation mode, arrays of 11T SRAM units enable large-scale parallel computing capabilities to be achieved. Via the integrated aXNOR computation transistor structure, they perform dynamic analog Multiply–Accumulate (MAC) operations on external input signals X and internally stored weight data W. When receiving the real-time varying external input signal X, each 11T SRAM cell immediately performs parallel computation against its stored weight W; this fuses the storage and computation functions to generate a unit current output that is directly proportional to the MAC operation on the pair (X, W). Before delving into a detailed analysis of the MAC current generation, let us discuss the re-encoding logic applied to Feature port input signals. The proposed analog multiplication design redefines the binary representation of the complementary signal pair {X, XB}: when {X, XB} takes the value {1, 0}, the Feature signal represents a positive attribute with a Feature value of +1; conversely, when {X, XB} is {0, 1}, the Feature value is defined as −1. Similarly, the Weight storage values in the DAM SRAM cell adopt a comparable binary redefinition strategy, where the complementary signal pair {Q, QB} yields a Weight value of +1 when {Q, QB} is {1, 0}, and a weight value of −1 when {Q, QB} is {0, 1}.

**Figure 4:**
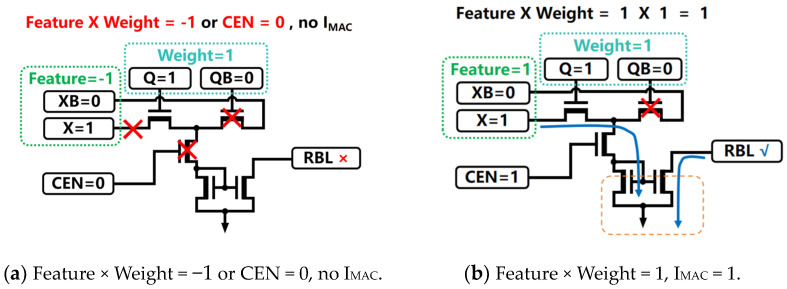

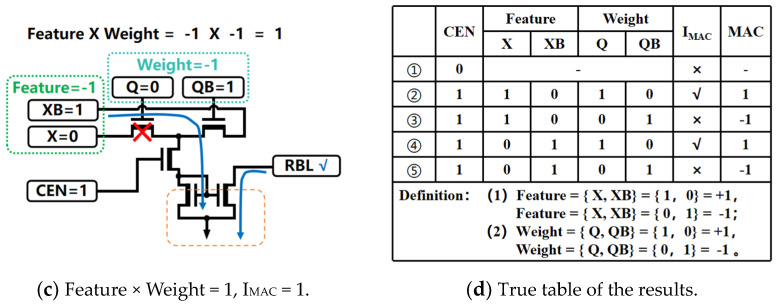
In academic circuitry terms, each DAM SRAM cell admits four distinct configurations with respect to external input signals, with each configuration yielding a unique multiplication outcome.

In the SRAM storage mode, the dynamic aXNOR enable signal is passed through the COM_EN port, setting the CEN signal to 0; this causes the MOSFET controlled by the CEN to be turned off, thus preventing any effective current path from being established and ensuring that the DAM SRAM unit does not execute any analog MAC computations. However, upon switching to the computation mode, the CEN signal is set to 1; this turns on the MOSFET and allows the Feature input signal X to interact with the Weight signal Q stored in the DAM SRAM cell for analog multiplication. As shown in Figure 4, four distinct signal configuration combinations exist, and each corresponds to a unique multiplication result.

(1) When the Feature signal pair {X, XB} has a value of {1, 0} (corresponding to +1) and the Weight signal pair {Q, QB} has a value of {1, 0} (also +1), the analog multiplication produces a result of +1, generating a mirrored current IC that is proportional to the analog MAC current IM on RBL.

(2) If the Feature signal pair {X, XB} has a value of {0, 1} (representing −1) and the Weight signal pair {Q, QB} has a value of {0, 1} (also −1), although both are negative, their product is still +1, resulting in a mirrored current IC on RBL.

(3) When the Feature signal pair {X, XB} has a value of +1 and the Weight signal pair {Q, QB} has a value of −1, the analog multiplication result becomes −1, theoretically leading to no generation of an analog multiplication result current; hence, no corresponding mirrored current is observed on RBL.

(4) Lastly, if the Feature signal pair {X, XB} has a value of −1 and the Weight signal pair {Q, QB} has a value of +1, the analog multiplication again results in −1; similarly, no reflected analog current would represent the multiplication outcome on RBL.

The truth table of the 11T SRAM cell exhaustively records all possible combinations of the input signal X and weight data W, as well as the corresponding unit current output values for each combination.

### 2.2. The Voltage Gradient Quantization Circuit

To address the non-linearity issues [6] that arise from accumulated analog currents, this design further incorporates a voltage gradient quantization circuit. In practical operations, the MAC results in the manifestation of the analog domain as accumulated analog currents, which, if left unprocessed, may introduce non-linearity into the output current and compromise the accuracy of the final computational results due to source voltage drift and other factors. The voltage gradient quantization circuitry converts these analog currents in real-time into stable voltage gradients, ensuring a high level of output precision throughout the computational process.

**Figure 5:**
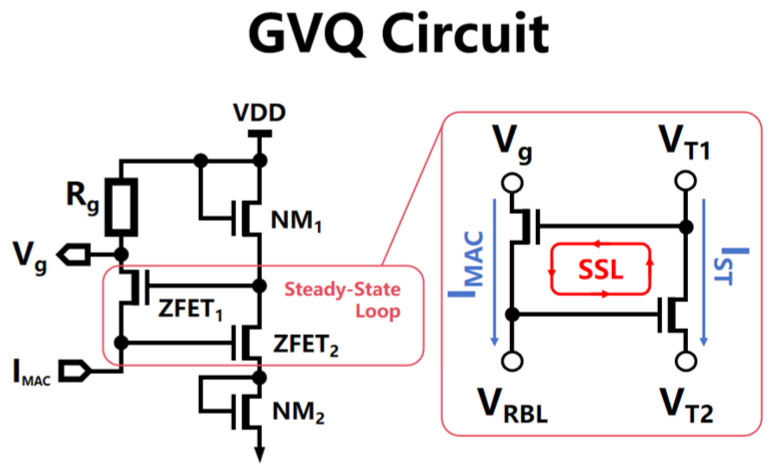
The voltage gradient quantization circuit consists of a quantified resistance (Rg), a set of partial voltage MOS tubes (NM 1 and NM 2), and a steady-state loop (SSL, ZFET 1, and ZFE 2). The input is the MAC current IMAC for the current accumulation bus RBL, and the output is the gradient quantization voltage of the quantified resistance to the IMAC.

A system consisting of 64 voltage gradient quantization circuit units, designed based on the aXNOR-based in-memory computing principle of the DAM SRAM array, is arranged in a matrix-like column layout, closely aligned and corresponding one-to-one with each column in the DAM SRAM array. This array efficiently translates the large-scale analog cumulative currents that are generated during the parallel distributed computation of the DAM SRAM array into a high-fidelity sequence of voltage gradient signals. As shown in Figure 5, each voltage gradient quantization circuit consists of a quantization resistor (Rg), a set of voltage divider MOSFETs (NM1 and NM2), and a self-stabilizing loop (SSL, ZFET1, and ZFET2. During multi-row MAC operations, individual memory cells within the same column perform multiplication with their stored states against the accumulating result, with the resultant currents progressively summed up on the RBL; this eventually forms voltage changes that are representative of data states and appear as voltage drops across the RBL and its complementary line RBLB. As an increasing number of inputs participate in the MAC operations and their corresponding weight coefficients, the signal margin of the system exhibits a marked exponential tendency to decay, which affects the accuracy of the analog readout when the margin falls below the inherent offset threshold of the analog readout circuits, sense amplifiers (SAs), or analog-to-digital converters (ADCs).

**Figure 6:**
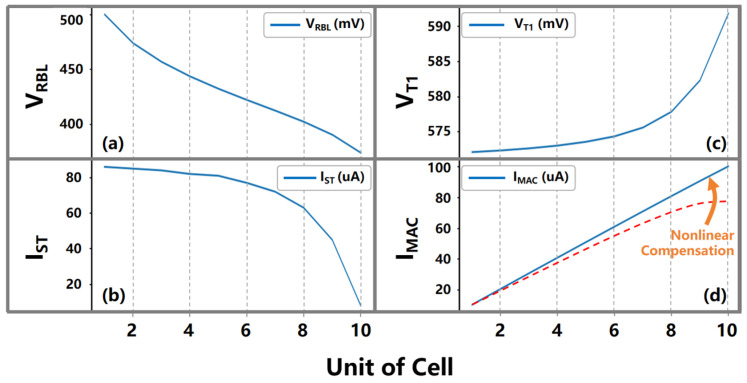
(**a**–**d**) The proposed voltage gradient quantization circuit addresses the non-linear attenuation issue of voltage gradients caused by the current accumulation effect on RBL through a self-stabilizing operating mechanism. While the self-stabilizing loop does not directly solve for the voltage drift phenomenon on the RBL current accumulation bus, it compensates for the non-linearity of the MAC currents by elevating the bias voltage of ZFET1.

The voltage gradient quantization circuit proposed in this paper addresses the non-linear attenuation issue of voltage gradients caused by the effect of current accumulation on RBL through a self-stabilizing operating mechanism. While the self-stabilizing loop does not directly solve for the voltage drift phenomenon on the RBL current accumulation bus, it compensates for the non-linearity of the MAC currents by elevating the bias voltage of ZFET1 via the following steps: (1) As shown in Figure 6a, as the number of contributing inputs and their corresponding weight coefficients increase during MAC operations, the non-linear attenuation effect of the accumulated current intensifies; this causes a non-linear drift in the voltage VRBL on the current accumulation bus RBL, as depicted by the dashed line in Figure 6d. (2) The bias voltage of ZFET2 originates from the voltage VRBL on the current accumulation bus RBL. As shown in Figure 6b, when the VRBL undergoes non-linear drift downward, the current IST in the right half of the self-stabilizing circuit branch decreases correspondingly, which raises the source voltage VT1 of ZFET2, as shown in Figure 6c. (3) As illustrated in Figure 6a, the bias voltage of ZFET1 originates from the source voltage VT1 of ZFET2. When VT1 is increased, the MAC current IMAC on the current accumulation bus RBL receives non-linear compensation, maintaining a linearly increasing trend, as depicted in Figure 6d; this solves the problem regarding the non-linear attenuation of the output voltage gradient Vg. The gradient voltage signals processed by the voltage gradient quantization circuits are fed into a gradient voltage encoding module, which meticulously analyzes and accurately quantizes these signals, thereby generating multi-bit-wide digital output signals. This voltage gradient quantization circuit significantly enhances the ability of the in-memory computing system to perform linearization for analog current accumulation results, thereby improving the overall computational accuracy and long-term stability of the system.

### 2.3. The Pre-Processing Strategy of Data Storage

**Figure 7:**
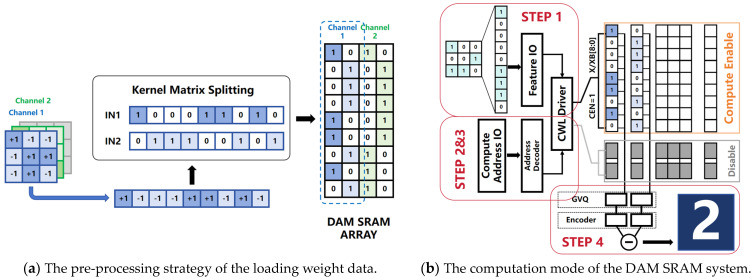
Before executing a convolutional layer operation, the DAM SRAM core enters the SRAM mode, loading image-relevant weight data column-wise into the DAM SRAM core array. A pre-processing strategy in which the convolution kernel is vectorized and decomposed is proposed. After the weight loading phase, the DAM SRAM system transitions from the storage mode to the computation mode.

Before executing a convolutional layer operation, the DAM SRAM core enters the SRAM mode, loading image-relevant weight data column-wise into the DAM SRAM core array. Both the weights stored in the DAM SRAM cells and input signals are represented in binary format, consisting only of 1 s and 0 s; meanwhile, the original convolution kernel elements use signed binary values of +1 and −1. A pre-processing strategy, depicted in Figure 7a, is proposed; here, the convolution kernel is vectorized and decomposed. Using the mathematical transformation Kernel = IN1 − IN2, the kernel is converted into two complementary parts, IN1[8:0] and IN2[8:0], to meet the operational requirements of the DAM SRAM core. These two subsets, IN1 and IN2, are independently fed into the DAM SRAM array in a nine-line parallel manner and stored in nine consecutive rows within the same column, for example, rows 8 to 0; this is followed by the performance of synchronous weighted accumulation operations based on XOR logic upon input data. After the weight loading phase, the DAM SRAM system transitions from the storage mode to the computation mode. The overall computational process is detailed in Figure 7b.

The first step involves the input of feature data F [8:0] from external I/O interfaces and the performance of pre-processing by the CWL driver circuitry. This circuit converts the feature data F [8:0] into a pair of complementary binary sequences, namely X [8:0] and XB [8:0], which are then separately sent to the DAM SRAM array to match the rows storing IN1 and IN2, respectively. In the second step, the CWL driver circuitry, along with the address decoder, assigns addresses to X [8:0] and XB [8:0]; this spatially pairs the complementary binary data sequences {X [8:0], XB [8:0]} with the corresponding two parts of the decomposed convolution kernel {IN1[8:0], IN2[8:0]} stored in the DAM SRAM array. The third step sees the simultaneous activation of the computation enable signals for rows 8 through 0. Setting the CEN signal high via the COM_EN port triggers the execution of MAC operations in the respective rows, thus performing row-wise multiplication and accumulation operations between {X [8:0], XB [8:0]} and IN1[8:0] and IN2[8:0]. Finally, the current-based results obtained from the Multiply–Accumulate operations are converted into digital outputs via integrated voltage gradient quantization and the gradient voltage decoding circuits. The subtraction of these digital values yields the final four-bit digital output result O [3:0].

### 2.4. The ADC and Output Stage

**Figure 8:**
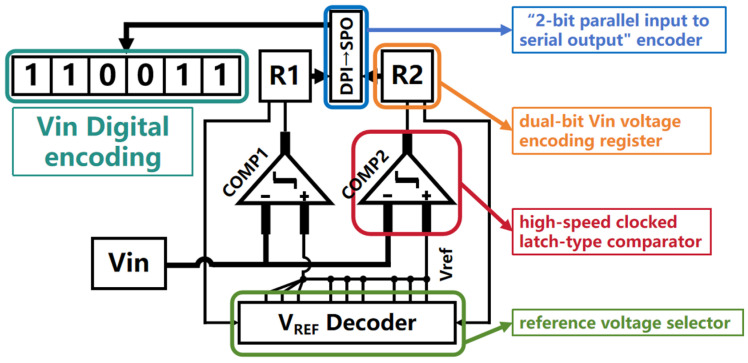
In the present design, the implementation of a gradient voltage encoding circuit is employed for both the ADC and output stage, comprising two high-speed clocked latch-type comparators, a dual-bit Vin voltage encoding register (R1 and R2), a reference voltage selector, and a “2-bit parallel-to-serial output” encoder.

As shown in Figure 8, the implementation of a gradient voltage encoding circuit is employed for both the ADC and output stage, comprising two high-speed clocked latch-type comparators, a dual-bit Vin voltage encoding register (R1 and R2), a reference voltage selector, and a “2-bit parallel-to-serial output” encoder. The high-speed clocked latch-type comparators serve to output the comparison result between the selected reference voltage and Vin during each cycle. The Vin voltage encoding registers, R1 and R2, temporarily store the comparator outputs and provide voltage selection enable signals to the reference voltage selector. The reference voltage selector utilizes the voltage selection signals from the encoding registers to choose the appropriate reference voltage for the high-speed comparators. Lastly, the “2-bit parallel-to-serial output” encoder converts the numerical values stored in the voltage encoding registers into a serial output format.

At the onset of a complete conversion cycle within the gradient voltage encoding circuit, the Vin voltage encoding registers undergo reset and initialization. During this initialization process, all bits within the registers adhere to a binary-split principle, configuring themselves to an intermediate reference voltage level encoding value that is symmetrically distributed relative to the full-scale reference voltage. This intermediate level corresponds to one-half of the quantized reference voltage range, effectively presetting an initial reference voltage. This preconfiguration serves to swiftly bracket the likely dynamic range of the analog input voltage Vin during the subsequent analog-to-digital conversion process. Once the initial reference voltage benchmark is set in the Vin voltage encoding registers, the two high-speed clocked latch-type comparators commence alternating operations. Using the reference voltage selected during initialization, these comparators periodically compare the target analog input voltage Vin. If the comparison indicates that Vin is higher than the initially locked reference voltage threshold, the state-holding register R1 maintains a logical high state (“1”), signifying that the current reference voltage estimate is lower than the actual Vin. Consequently, the reference voltage Vref needs to be incremented towards a higher voltage domain. Conversely, if Vin is lower than the chosen reference voltage Vref, R1 is driven to a logical low state (“0”), indicating that the current Vref selection is too high and requires downward adjustment. With each clock pulse, Vin is successively compared against COMP1 and COMP2 comparator units in an alternating fashion. The resulting comparison outputs drive alternating updates in the states of R1 and R2. Based on the previous state decision made by R1, the reference voltage selection circuit selects the reference input voltage level for the next comparison cycle.

### 2.5. The Low-Power Design of DAM SRAM

The computation structure of the DAM SRAM unit, particularly the aXNOR portion, incorporates an in-unit current isolation mechanism that significantly reduces the system’s consumption of computational power. On one hand, this programmable in-unit current isolation mechanism enables the DAM SRAM array to perform flexible block dormancy, thereby decreasing the consumption of static power in the DAM SRAM CORE blocks that do not participate in computations. On the other hand, the high-speed dynamic nature of the in-unit current isolation mechanism effectively minimizes the consumption of computational power in DAM SRAM blocks during the computation mode, realizing the low-power operation of the DAM SRAM CORE.

**Figure 9:**
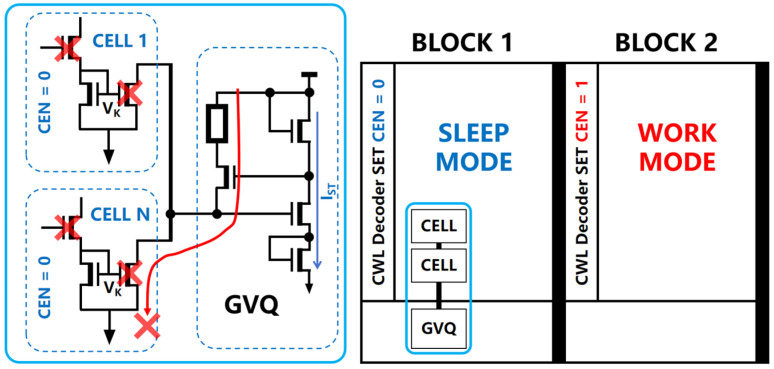
In the block dormancy mode, when Block 1 in the DAM SRAM array is instructed to enter dormancy, the entirety of Block 1’s COM_EN port sets the CEN signal to a low level, disabling the internal computational pathways of all DAM SRAM units within the block.

In the block dormancy mode, as shown in Figure 9, when Block 1 in the DAM SRAM array is instructed to enter dormancy, the entirety of Block 1’s COM_EN port sets the CEN signal to a low level, thus disabling the internal computational pathways of all DAM SRAM units within the block. The node voltage VK within the aXNOR structure is pulled down, preventing the formation of a current path in the left branch of the voltage gradient quantization circuit; this leaves only a small static current IST in the right branch, thus enabling the block to achieve a low-power sleep mode.

**Figure 10:**
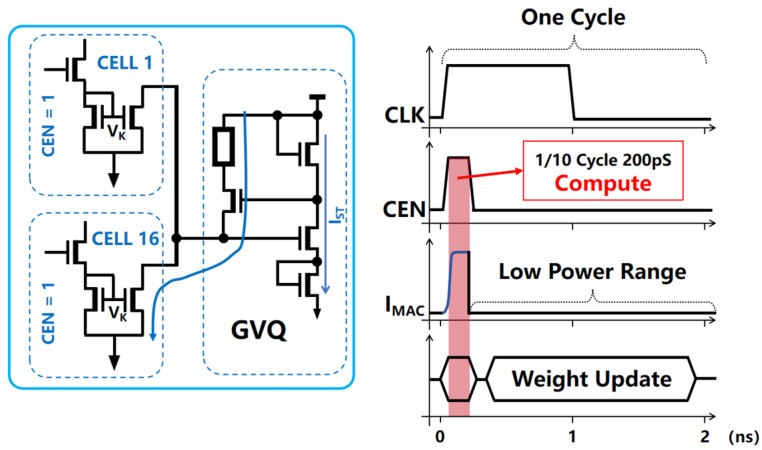
In order to conserve energy and prevent the unnecessary accumulation of power, the system dynamically controls the COM_EN port to revoke the high-level CEN signal during the calculation cycle; this restores it to a low level, thereby cutting off the computational current path in the left branch of the voltage gradient quantization circuit. By ensuring that the computational period is only 1/10th of the total cycle, the dynamic enabling signal can reduce the array’s consumption of computational power to merely 10%.

In the computation mode, when the DAM SRAM array performs computations, the COM_EN control ports corresponding to the rows with active data are prompted to set the CEN signal to a high level. This action activates the computational pathways within the DAM SRAM units of the engaged block. The node voltage VK within the aXNOR logic structure of each DAM SRAM unit rises to a preset higher voltage level, consequently activating the in-unit current mirror connected to it. The current mirror regulates the unit current intensity during analog computations. Simultaneously, the left branch of the voltage gradient quantization circuit forms a continuous current path, generating gradient voltages according to the state differences within the unit data. In the actual computation mode, the DAM SRAM array can simultaneously activate up to 16 independent rows for calculations. Assuming that all DAM SRAM units in a given row contribute a computational result of +1 during a calculation cycle, the left branch of the voltage gradient quantization circuit will accumulate basic computation currents from 16 units, resulting in a total current output of up to 160 uA. In order to conserve energy and prevent the unnecessary accumulation of power, the system dynamically controls the COM_EN port to revoke the high-level CEN signal during the calculation cycle; this restores it to a low level and thereby cuts off the computational current path in the left branch of the voltage gradient quantization circuit. As shown in Figure 10, by ensuring that the computational period is only 1/10th of the total cycle, the dynamic enabling signal can reduce the array’s consumption of computational power to merely 10%.

## 3. Analysis and Simulation

### 3.1. Binary Convolutional Neural Network [7] Computation

**Figure 11:**
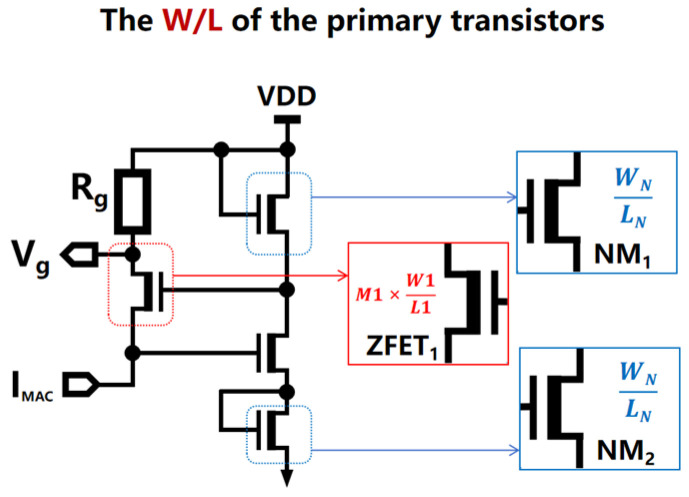
In order to conserve energy and prevent the unnecessary accumulation of power, the system dynamically controls the COM_EN port to revoke the high-level CEN signal during the calculation cycle; this restores it to a low level, thereby cutting off the computational current path.

In the proposed design, the voltage gradient quantization circuit utilizes a self-stabilizing voltage operating mechanism, which addresses the non-linear decay caused by the effects of RBL current accumulation; this allows the accumulated analog current signals to be accurately quantified and ensures that the gradient of the output voltage Vg remains linear, thereby mitigating the occurrence of non-linear decay phenomena. As shown in Figure 11, three critical parameters are considered in the voltage gradient quantization circuit: (1) the channel length (L1) of ZFET1, (2) the channel width (M1 and W1) of ZFET1, and (3) the aspect ratio (WN/LN) of NM1/NM2 transistors; these parameters have a significant impact on the performance of the circuit, including its quantization precision (computational linearity), consumption of static and computational power consumption per unit area, and circuit area.

ZFET represents a super-low-threshold voltage transistor, distinguished from conventional CMOS FETs by virtue of its significantly lower threshold of voltage, or turn-on voltage, and the ability to maintain stable currents even under minute variations in gate voltage. When the current in the left branch of the GVQ circuit becomes insufficient due to an inadequate number of computational units, ZFETs excel in enabling a rapid response from the GVQ circuit.

#### 3.1.1. The Channel Length (L1) of ZFET1

**Figure 12:**
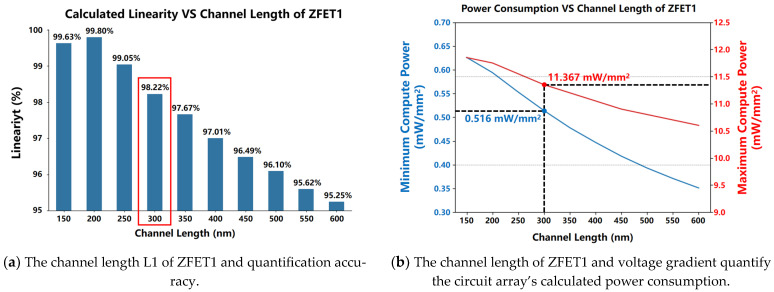
While increasing L1 improves the computational power consumption of the 64 × 1 array circuit, it concurrently decreases the precision and linearity of the accumulated current quantization.

Considering a unit channel width of 500 nm for ZEFT1, with M1 denoting a finger count of 20, and an aspect ratio of 120 nm/300 nm for the NM1/NM2 transistors, this study investigated the impact of varying the channel length L1 of the ZEFT1 transistor on the quantization precision and minimum (with only one computational unit current) to maximum (with eight computational units of current) computational power consumption of the voltage gradient quantization circuit. As depicted in Figure 12, while increasing L1 led to improvements in the minimum and maximum computational power consumption of the 64 × 1 array circuit, it simultaneously reduced the precision and linearity of the accumulated current quantization. Consequently, taking into account the circuit area, power consumption (minimum computational power < 0.55 mW/mm^2^, maximum computational power < 11.5 mW/mm^2^), and linearity (linearity > 98% with eight computational units), the channel length L1 of the ZEFT1 transistor was set at 300 nm.

In the experiment assessing calculation linearity, as per Figure 12a, channel lengths were divided into ten groups ranging from 150 to 600 nanometers. For each group, measurements were taken of the unit output current when the circuit was driven by an eight-unit computational current output, the IMAC current in the current accumulation bus, and the output voltage of the GVQ circuit. Calculation linearity was determined by calculating the ratio of the IMAC current in the accumulation bus to the theoretical total IMAC current. The specific data obtained are presented in Table 1.

#### 3.1.2. The Channel Width (M1 and W1) of ZFET1

**Figure 13:**
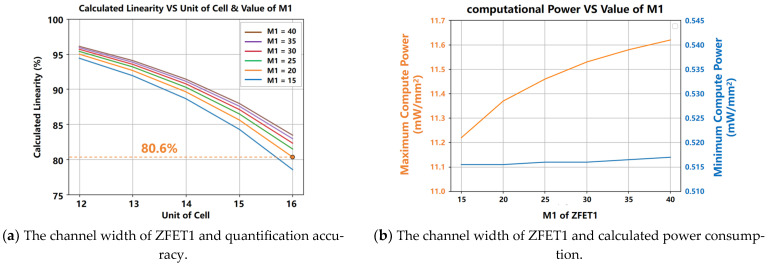
As M1 increases, under the same number of computing units, both the quantization precision and consumption of computational power per unit area increase. It is evident from the blue curve that, when there are 16 computing units, the quantization precision approaches 80%, and that at M1 = 29, the precision drops below 80%.

Owing to ZEFT1’s fixed channel length L1 of 300 nm and unit channel width W1 of 500 nm, the effect of changing the total channel width M1 × W1 via modifying the number of fingers (M1) on the precision of quantization and consumption of computational power per unit area was examined. As shown in Figure 13, with M1 increased, under the same number of computing units, both the quantization precision and consumption of computational power per unit area increased. As can be seen from the blue curve that, when there were 16 computing units, the quantization precision approached 80%; at M1 = 10, the precision dropped below 80%. To ensure the precision of the system while limiting the number of simultaneously computed units to 16 or fewer, M1 was set as 20. This balanced the increase in computational precision against the rising consumption of computational power per unit area.

#### 3.1.3. The Aspect Ratio (WN/LN) of NM1/NM2

**Figure 14:**
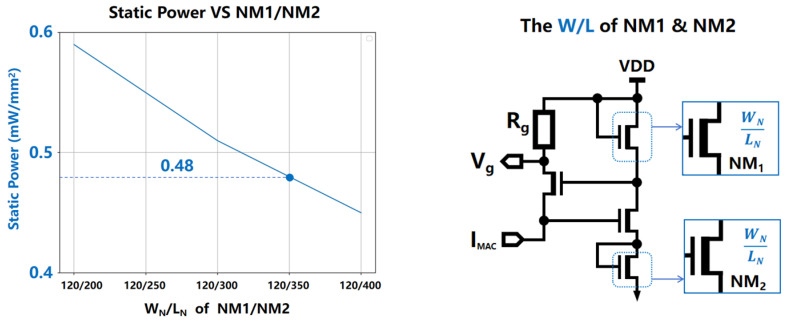
The aspect ratio WN/LN of NM1/NM2 determines the static current in the right branch array of the voltage gradient quantization circuit, hence affecting its consumption of static power per unit area.

The aspect ratio WN/LN of NM1/NM2 governs the static current within the right branch array of the voltage gradient quantization circuit, thereby influencing its static power consumption per unit area and the minimum computational power consumption. As shown in Figure 14, experimental observations revealed that WN/LN had a relatively minor impact on the static current, maintaining a range of between 100 nA and 105 nA. Consequently, the determination of WN/LN for NM1/NM2 was primarily guided by the minimum computational current. With the objective of maintaining the minimum computational power consumption at below 0.5 mW/mm^2^, WN/LN was set at 120/350. During the experiment aimed at measuring the minimum computational power consumption, the primary focuses were twofold: ensuring a power level below 0.5 mW/mm^2^ and minimizing the circuit area while preserving symmetry. Given that each column of the SRAM is equipped with a GVQ circuit, circuit area optimization became a crucial consideration, necessitating a compact design that would not compromise the desired power threshold. Simultaneously, maintaining symmetry in the circuit layout was deemed vital for both functional efficacy and manufacturability. Based on these dual objectives, an aspect ratio of 120/350 was selected as it effectively strikes a balance between achieving the targeted low minimum computational power consumption and retaining a compact, symmetrical circuit layout, thereby satisfying both requirements and ensuring the overall efficiency and practicality of the design.

Based on the data obtained from the above simulations, we compiled the final values of the three core parameters of the voltage gradient quantization circuit in Table 2. Concurrently, we made a comprehensive record of the circuit’s performance under these parameter settings, which is presented in Table 3.

### 3.2. Binary Convolutional Neural Network Computation Analysis

In order to perform convolutional layer computations in a Binary Neural Network using DAM SRAM, the following steps must be implemented: (1) Matrix Reshaping: A 5 × 5 feature input matrix and a 3 × 3 convolution kernel require 52 vector multiplications for convolution. Thus, the feature input matrix is reshaped into a sequence of one-dimensional vectors that have a dimension of 25 and that contain nine elements each; these are input into the array over 52 cycles for the performance of MAC operations with weight data (convolution kernel). This reshaping process is accomplished through software compilation. (2) Weight Data Writing and Address Space Mapping: The input directions of the reshaped feature matrix vectors are along WL, which necessitates the remapping of the storage address space for weight data (convolution kernels); this is from the BL direction to the WL direction in the DAM SRAM array. A 16-channel 3 × 3 convolution kernel is divided into 16 regions for storage, which are arranged in 16 columns, and the remapping process is performed via software compilation. (3) Feature Matrix Input and Convolution Operation: After reshaping, the feature input matrix vectors undergo full-column parallel MAC operations using each row block of the remapped weight data storage areas in each cycle. Because the remapped storage area for the convolution kernel is in a single row block, completing the convolutional layer computation for the entire feature matrix and all channels requires 52 cycles. The CWL driver circuits and address decoding circuits within the DAM SRAM handle the matrix input process.

### 3.3. Binary Convolutional Neural Network Computational Simulation

Our convolutional layer computational simulation experiment proceeded as follows: Two convolutional layer computations were simulated, namely Cov L1 and Cov L2; these involved 8064 and 516,096 MAC operations, respectively. A conventional Von Neumann architecture served as the control group, where the CPU performs the convolutional layer computations with data that are stored in SRAM memory (MEM) interfaced with a 32-bit bus; this requires three clock cycles for a single read/write operation. The test group used a CPU+MEM+DAM SRAM setup, where DAM SRAM operates with eight parallel cores at 500 MHz under CPU control; here, the convolution computations are executed by the DAM SRAM CORE. The results showed that using the DAM SRAM CORE in conjunction with the CPU reduced the movement of data by more than 95% and correspondingly decreased the consumption of power by 95% during convolutional layer computations. Meanwhile, speedup improvements of 97.2% for L1 and 99.72% for L2 were achieved. Table 4 presents a comparison with prior related work, demonstrating that due to the adoption of dynamic low-power computation modes and low-power computation unit designs, we achieved significantly greater energy efficiency compared to other in-memory computing designs.

## 4. Results

In this paper, a novel design for the DAM SRAM CORE in-memory computation IP core has been presented, encompassing the overall architecture, details of the implementation of the main modules, and comprehensive simulation performance metrics. The IP core, designed around a dynamic aXNOR-based computing scheme within SRAM cells, supports single-bit Multiply–Accumulate (MAC) operations and features several key innovations: (1) The development of an 11T SRAM cell that integrates both storage and computation capabilities, enabling the performance of large-scale parallel MAC operations within the memory SRAM array. This cell demonstrates an efficient utilization of area and a compact layout. (2) The implementation of a dynamic aXNOR computation mode, which significantly reduces both the computational and static power consumed by the array. (3) The design of a self-stabilizing voltage gradient quantization circuit that enhances the system’s operational accuracy. Numerous simulations were performed using the 64 × 64-bit DAM SRAM CORE in-memory computation IP core, which was fabricated using a 55 nm CMOS logic process; these substantiated the efficacy of the proposed design. Under conditions of 1-bit input feature data, 1-bit weight data, and 5-bit output results, this chip exhibited low-power operation with a static power dissipation of 0.48 mW/mm^2^ and a computational power consumption of 11.367 mW/mm^2^. Furthermore, it achieved a data throughput of 109.75 GOPS and an energy efficiency of 21.95 TOPS/W, demonstrating its potential for application in high-performance, energy-efficient computing.

## Figures and Tables

**Table 1:** Calculating linearity and the channel length of ZFET1.

Unit Current (uA)	Channel Length	MAC Current (uA)	Theoretical MAC Current (uA)	Linearity(%)
I_0_	L/n	I_MAC_	I_MAC_L_	
8.24	150	66.1	65.92	99.63
	200	65.8		99.80
	250	65.3		99.05
	300	64.75		98.22
	350	64.39		97.67
	400	63.95		97.01
	450	63.61		96.49
	500	63.35		96.10
	550	63.03		95.62
	600	62.79		95.25

**Table 2:** The parameter values of the SSL.

Parameter	ZFET1		ZFET2		NM1/NM2
	W1/L1	M1	W2/L2	M2	WN/LN
Parameter Values	500 n/300 n	20	500 n/200 n	5	120 n/350 n

**Table 3:** The performance of the SSL.

Performance	P_ST_ (mW/mm^2^)	P_MAC_ (mW/mm^2^)	Linearity (%)	
Unit of Cell	-	8	8	16
Performance Value	0.48	11.367	98.22	80.60

**Table 4:** A comparison with other work.

	ISSCC 2020 [8]	JSSC 2021 [9]	ISSCC 2021 [10]	Electronics 2024 [11]	This Work
Technology	28 nm	65 nm	28 nm	65 nm	55 nm
Bit Cell(T)	6	6	6	9T1C	11
Array Size	64 Kb	512 × 256	384 Kb	32 × 32	64 × 64
Frequency	120 MHz	-	138 MHz	50 MHz	50 MHz
Bit Precision (Input/Weight/Output)	8/8/20	4/1/7	8/8/20	4/4/7	1/1/5
Throughput (GOPS)	30.48	573.4	-	102.4	109.75
Energy Efficiency(TOPS/W)	16.63	49.4	15.02~22.75	33.6	21.95

## Data Availability

Data are contained within this article.
